# Echocardiographic Evaluation for Safe and Complete Transcatheter Left Atrial Appendage Closure

**DOI:** 10.1016/j.jaccas.2025.106289

**Published:** 2025-12-04

**Authors:** Mizuki Miura, Akihiro Isotani, Masato Fukunaga, Kent Kito, Masataka Arakawa, Yusuke Watanabe, Ken Kozuma, Akihisa Kataoka

**Affiliations:** a Department of Medicine, Division of Cardiology, Teikyo University School of Medicine, Tokyo, Japan; b Department of Cardiology, Kokura Memorial Hospital, Kitakyushu, Japan

**Keywords:** echocardiography, mitral valve, ultrasound

## Abstract

**Objective:**

To demonstrate transcatheter left atrial appendage (LAA) closure guided by an expanded transesophageal echocardiography checklist, the CLOSE-PML sign (*c*ircumflex artery, *l*obe compression, *o*rientation, *s*eparation, *e*lliptical disc, plus *p*ulmonary structures [pulmonary artery and veins], *m*itral valve, *l*eak), to optimize Amplatzer Amulet occluder positioning and enhance procedural safety and efficacy.

**Key Steps:**

Beyond the standard CLOSE sign, PML adds: 1) pulmonary structures: confirm unobstructed left upper pulmonary vein flow and ≥1.5-mm clearance from the pulmonary artery; 2) mitral valve: verify normal leaflet motion and absence of new mitral regurgitation; and 3) leak: exclude substantial peridevice leak on color Doppler, confirming complete LAA sealing.

**Potential Pitfalls:**

Neglecting PML risks left upper pulmonary vein obstruction, pulmonary artery injury with delayed tamponade, new mitral regurgitation, and residual leaks predisposing to thrombus.

**Take-Home Message:**

Integrating PML with the CLOSE sign heightens intraprocedural vigilance, ensures systematic assessment of device-structure interactions, and promotes effective, durable transcatheter LAA closure.

## Visual Summary

**Figure:**
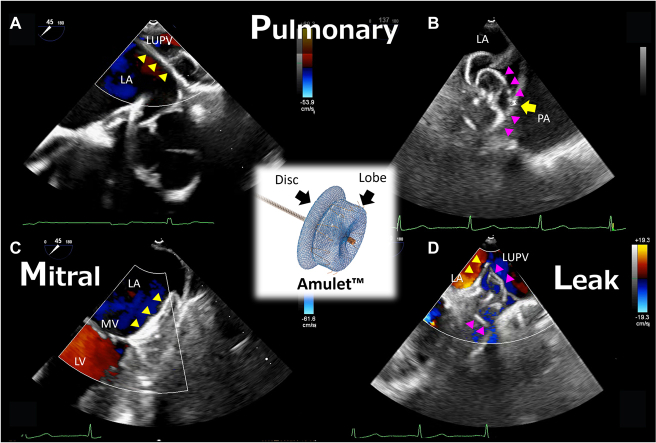

**Visual Summary:**
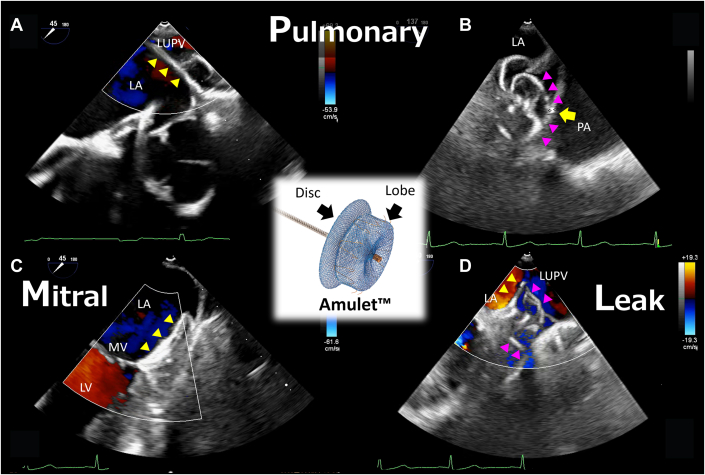
PML Assessment After CLOSE (CLOSE-PML Sign) (Center) Illustration of the Amplatzer Amulet occluder. (A to D) Transesophageal echocardiography images. (A) The disc (yellow triangle) obstructing flow in the left upper pulmonary vein (Video 4). (B) The distance between the left atrial appendage wall at the lobe (magenta triangle) anchor and pulmonary artery is 2.5 mm (arrow; the recommended distance is ≥1.5 mm) (Video 5). (C) The disc does not interfere with the mitral valve or cause regurgitation (Video 6). (D) There is no significant residual leak around the lobe or disc (Video 7). CLOSE = circumflex artery, lobe compression, orientation, separation, elliptical disc; LA = left atrium; LAA = left atrial appendage; LUPV = left upper pulmonary vein; LV = left ventricle; MV = mitral valve; PA = pulmonary artery, PML = pulmonary structures (pulmonary artery and veins), mitral valve, and leak.

Transcatheter left atrial appendage (LAA) closure is increasingly recognized as a viable alternative for stroke prevention in patients with nonvalvular atrial fibrillation and elevated thromboembolic risk who have a contraindication to or are not candidates for long-term oral anticoagulation.^1^ The Amplatzer Amulet occluder (Abbott Medical) is one of the most widely used devices for this purpose, and transesophageal echocardiography (TEE) plays a pivotal role in procedural guidance and confirmation of device positioning.

**Take-Home Messages**

• The CLOSE-PML sign extends the standard CLOSE evaluation by incorporating assessment of the pulmonary artery and pulmonary veins, mitral valve, and residual leak, thereby enhancing intraprocedural safety.
• Preventing device-related pulmonary artery impingement requires both careful preprocedural case selection and meticulous intraprocedural TEE assessment.

Although transcatheter LAA closure has demonstrated procedural safety and efficacy, late complications have been reported, including delayed pericardial effusion, which can be life-threatening and has been linked to device-related factors.^2^^,^^3^ One proposed mechanism of complication is device-related pulmonary artery impingement (DR-PAI), in which the distal lobe of the device lies in close proximity to the pulmonary artery, potentially causing chronic mechanical irritation or injury.^4^ In this case report, we discuss the importance of meticulous preprocedural imaging, intraprocedural vigilance, and comprehensive criteria for device release. In particular, while the standard CLOSE sign (circumflex artery, lobe, compression, separation, and elliptical disc) is useful to ensure appropriate device position, additional evaluation of the pulmonary structures (pulmonary artery and veins), mitral valve, and residual leak (PML) may provide a more robust framework for optimizing outcomes and preventing delayed complications.

## Case Summary

The patient was a 72-year-old man with persistent atrial fibrillation, dyslipidemia, and diabetes mellitus. He was transferred with decompensated heart failure, and subsequent ischemic evaluation demonstrated coronary artery disease, leading to percutaneous coronary intervention. Given a CHA_2_DS_2_-VASc score of 4, he was administered oral anticoagulation therapy, but he developed a cerebellar hemorrhage, requiring cessation of oral anticoagulation. He was referred for transcatheter LAA closure in view of a contraindication for long-term anticoagulation.

**Figure 1:**
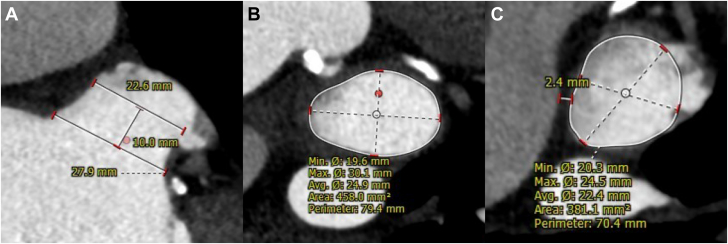
Cardiac CT Imaging for LAA Screening Cardiac CT revealed (A) a small, chicken wing–shaped LAA with sufficient depth for device deployment and no evidence of thrombus. The LAA measured (B) 30.1 × 19.6 mm at the ostium and (C) 24.5 × 20.3 mm at the planned landing zone. (C) At the landing zone, the distance between the pulmonary artery and the LAA was 2.4 mm. CT = computed tomography; LAA = left atrial appendage.

In accordance with the standard approach at our institution, the patient underwent cardiac computed tomography (CT) for preprocedural planning. Cardiac CT revealed a small, chicken wing–shaped LAA with enough depth to deploy the device, and no thrombus (Figure 1A). The LAA dimensions were 30.1 × 19.6 mm at the ostium (Figure 1B) and 24.5 × 20.3 mm at the estimated landing zone (Figure 1C). At the estimated landing zone, the distance between the pulmonary artery and the LAA was 2.4 mm (Figure 1C).

## Procedural Steps

**Figure 2:**
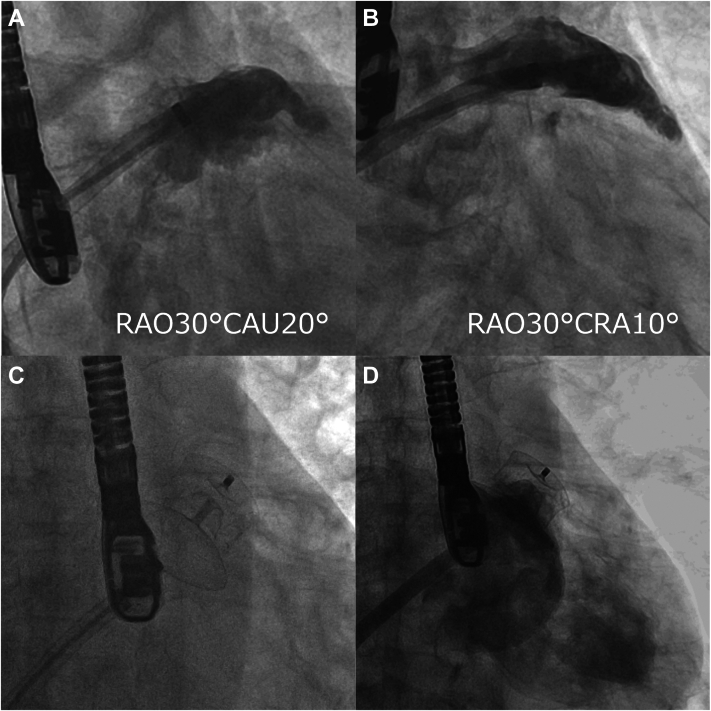
Angiography During the Procedure (A and B) The LAA was in its anticipated position and of the anticipated size, with clear visualization of pectinate muscle development and distribution (Video 1, Video 2, respectively). (C) During the tension test, device anchoring was confirmed by gently pulling on the delivery cable (Video 3). (D) Final angiography showing the LAA in the expected location and of the expected size, and no peridevice leak (Video 8). LAA = left atrial appendage.

The transcatheter LAA closure procedure was performed under general anesthesia with fluoroscopy and TEE guidance. Transseptal puncture through the fossa ovalis was attempted with a standard 63-cm 8-F SL0 sheath (Abbott Medical) together with a 71-cm RF needle C0 curve (Boston Scientific). After the transseptal puncture, an Amplatzer Amulet delivery sheath (Abbott Medical) was inserted across the atrial septum over a SupersStiff wire (Boston Scientific), and a 6-F pigtail catheter was introduced with a delivery sheath. Angiography revealed the LAA in the expected location and of the expected size, with development and distribution of the pectinate muscles (Figures 2A and 2B, Videos 1 and 2, respectively). Based on intraprocedural TEE measurements of the ostium and landing zone, a 28-mm Amplatzer Amulet device was used. The lobe portion was released first, inside the LAA body. Once the lobe was stable, the sheath was retracted to allow the disc to expand and cover the LAA ostium. Proper anchoring was ensured by gently pulling on the delivery cable to confirm stability without dislodgement, known as the “tension test” (Figure 2C, Video 3).

Before final device release, interventional cardiologists rely on echocardiographic evaluations such as the CLOSE sign, a checklist of key factors confirming correct device positioning prior to release.^5^ Specifically, the CLOSE sign encompasses (C) correct lobe position, typically with ≥2/3 of the lobe beyond the left circumflex artery; (L) adequate compression of the device's lobe, indicating proper sizing and secure wall apposition; (O) proper orientation of the lobe coaxial with respect to the LAA neck's axis; (S) clear separation between the occluder's disc and lobe; and (E) an elliptical (concave) disc shape, confirming that the disc is expanded and covering the ostial rim. Adhering to the CLOSE sign is crucial for procedural success, to ensure complete LAA sealing and device stability, thereby minimizing residual leaks and thrombus formation.^5^ Although the standard CLOSE sign addresses Amulet occluder positioning, some important aspects are not explicitly covered. To address aspects not covered by the CLOSE sign, we propose an expanded checklist, adding additional assessment of the pulmonary structures (pulmonary artery and veins), the mitral valve, and residual leak (PML)—CLOSE-PML—as described below.

• Pulmonary vein and artery: Ensure that the Amulet disc does not obstruct flow in the left upper pulmonary vein behind the LAA ridge. This includes inspecting color Doppler images (Video 4). Additionally, to avoid pulmonary artery injury that can cause late-phase cardiac tamponade, the distance between the LAA wall at the device anchor and the pulmonary artery should be at least 1.5 mm (Video 5).
• Mitral valve: Confirm normal mitral valve motion, and coaptation and separation from the disc. This is typically done by examining TEE views of the mitral valve (eg, 0°, 45°, or 90°) with color Doppler after device placement but prior to release.^6^ No new mitral regurgitation or contact with the mitral valve should be caused by the presence of the disc (Video 6).
• Leak: Ensure that there is no substantial residual leak around the lobe or disc, and that the LAA neck and ostium are completely sealed. In a previous study, a peridevice leak of ≥3 mm was associated with a significantly increased risk of stroke, systemic embolism, or cardiovascular death in the late phase.^7^ Although the original CLOSE sign implicitly aims for complete occlusion, the “L” in PML reiterates the need to thoroughly determine whether there is any peridevice flow on color Doppler (Video 7).

**Figure 3:**
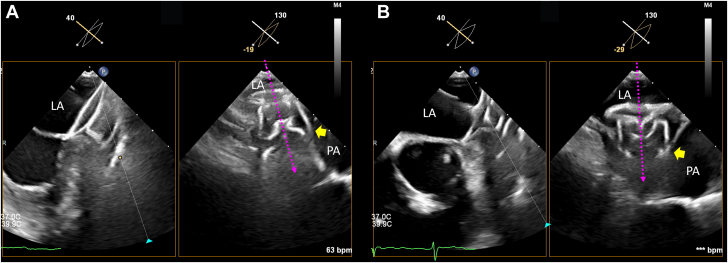
TEE Assessment for Pulmonary Artery (A) Initial deployment oriented the lobe tip toward the 5 o'clock position on a 130° TEE view (magenta dotted arrow). The LAA wall pressed against the pulmonary artery at the level of the distal lobe anchors (yellow arrow), increasing the contact surface between the anchors and the artery and raising concern for DR-PAI (Video 5). (B) After recapturing the device, the second deployment rotated the tip toward the 6 o'clock position at 130° (magenta dotted arrow), thereby reducing pulmonary artery contact and mitigating impingement risk (yellow arrow) (Video 6). DR-PAI = device-related pulmonary artery impingement; LA = left atrium; LAA = left atrial appendage; PA = pulmonary artery; TEE = transesophageal echocardiograph.

After assessment with CLOSE-PML, angiography should be conducted to confirm an LAA in the expected location and of the expected size, and to confirm no peridevice leak (Figure 2D, Video 8). The Amulet device is then released. In the current case, during the initial deployment the lobe tip was oriented toward the 5 o'clock position (more anterior) on TEE at 130°, resulting in a larger contact surface between the distal lobe anchors and the pulmonary artery, as well as a reduced distance to the vessel. This raised concerns regarding the risk of DR-PAI (Figure 3A, Video 9). Consequently the device was recaptured, and during the second deployment the axis was adjusted such that the lobe tip was oriented more toward the 6 o'clock position on TEE at 130° (Figure 3B, Video 10), thereby minimizing the contact surface between the anchors and the pulmonary artery in an effort to mitigate the risk of DR-PAI.

## Potential Pitfalls

Our approach facilitates smooth and successful transcatheter LAA closure with the Amplatzer Amulet, minimizing the risk of complications. Several key points that enable this are highlighted below.

### Intraprocedural imaging

For other transcatheter LAA closure devices such as the Watchman (Boston Scientific), the PASS (position, anchor, size, and seal) criteria are used for prerelease evaluation of the device. While all components of the PASS criteria must be assessed by TEE or intracardiac echocardiography, all but one of the CLOSE components can be evaluated by fluoroscopy. The exception is the “C” component, which requires confirmation that more than two-thirds of the lobe is positioned distal to the left circumflex artery. Adding PML assessment using TEE to the standard CLOSE evaluation further elevates intraprocedural vigilance, helping to ensure effective transcatheter LAA closure, and possibly to improve patient outcomes.

### Device-related pulmonary artery impingement

Delayed pericardial effusion has been reported after transcatheter LAA closure with the Amplatzer Amulet device, and it has been associated with adverse outcomes.^2^^,^^3^ One potential mechanism is device-related pulmonary artery interference or injury owing to the close anatomical relationship between the LAA wall and the pulmonary artery.^4^ To prevent DR-PAI, careful attention should be paid to the distance between the distal lobe position and the pulmonary artery as estimated on preprocedural CT or TEE, and patients with extremely close proximity should be screened out to ensure appropriate case selection. In addition, intraprocedural assessment using TEE is essential, including thorough evaluation of the “P” component of the PML sign. As well as the pulmonary artery distance, awareness of the contact surface and contact angle between the distal lobe anchors and the pulmonary artery, and minimizing these factors, may help to prevent DR-PAI.

## Conclusions

Incorporating the CLOSE-PML sign into intraprocedural evaluation provides a more comprehensive framework for optimizing device positioning and minimizing complications during Amplatzer Amulet transcatheter LAA closure. Careful preprocedural screening combined with vigilant intraprocedural imaging may help to prevent DR-PAI and improve long-term patient outcomes.

## Funding Support and Author Disclosures

Drs Isotani and Fukunaga have received consulting fees from Abbott Medical Japan as proctors for the Amplatzer Amulet occluder. In addition, the Department of Medicine, Division of Cardiology, Teikyo University School of Medicine has received a scholarship donation from Abbott Medical Japan. All other authors have reported that they have no relationships relevant to the contents of this paper to disclose.

## Appendix

Video 1 LAA Angiography in the RAO 30°/Caudal 20° View Demonstrating the LAA Anatomy, Corresponding Approximately to a 135° View on TEE. LAA = left atrial appendage; RAO = right anterior oblique; TEE = transesophageal echocardiography.

Video 2 LAA Angiography in the RAO 30°/Cranial 10° View Demonstrating the LAA Anatomy, Corresponding Approximately to a 45° View on TEE. LAA = left atrial appendage; RAO = right anterior oblique; TEE = transesophageal echocardiography.

Video 3 Tension Test Device anchoring was confirmed by gently pulling on the delivery cable.

Video 4 Color Doppler TEE Showing the Ostium of the Left Upper Pulmonary Vein A protruding Amulet disc obstructs blood flow in the left upper pulmonary vein. TEE = transesophageal echocardiography.

Video 5 TEE Image Showing the Amulet Device Deployed in the LAA The pulmonary artery was visualized in proximity to the lobe anchor. LAA = left atrial appendage; TEE = transesophageal echocardiography.

Video 6 Color Doppler TEE Showing the Amulet Disc Adjacent to the Mitral Valve Mitral regurgitation was not observed. TEE = transesophageal echocardiography.

Video 7 Color Doppler TEE Image of the Amulet Device in the LAA No substantial residual leak was observed around the lobe or disc. LAA = left atrial appendage; TEE = transesophageal echocardiography.

Video 8 Final Angiography An LAA in the expected location and of the expected size, with no peridevice leak. LAA = left atrial appendage.

Video 9 Biplane TEE Image of the Amulet Device in the LAA The LAA wall pressed against the pulmonary artery at the level of the distal lobe anchors. LAA = left atrial appendage; TEE = transesophageal echocardiography.

Video 10 Biplane TEE Image of the Amulet Device in the LAA After recapturing the device, the second deployment rotated the tip toward the 6 o'clock position at 130°. LAA = left atrial appendage; TEE = transesophageal echocardiography.
